# Antibacterial and Antifungal Properties of Modified Chitosan Nonwovens

**DOI:** 10.3390/polym14091690

**Published:** 2022-04-21

**Authors:** Dominik Sikorski, Marta Bauer, Justyna Frączyk, Zbigniew Draczyński

**Affiliations:** 1 Institute of Textile Materials and Polymer Composites, Lodz University of Technology, Zeromskiego 116, 90-924 Lodz, Poland; zbigniew.draczynski@p.lodz.pl; 2 Department of Inorganic Chemistry, Faculty of Pharmacy, Medical University of Gdańsk, 80-416 Gdańsk, Poland; marta.bauer@gumed.edu.pl; 3 Institute of Organic Chemistry, Lodz University of Technology Faculty of Chemistry, Zeromskiego 116, 90-924 Lodz, Poland; justyna.fraczyk@p.lodz.pl

**Keywords:** chitosan salts, bacteriostatic, acids

## Abstract

Chitosan acquires bacteriostatic properties via protonation of its amino groups. However, much of the literature assumes that chitosan itself inhibits the growth of bacteria. This article presents a comparative study of chitosan nonwovens modified with various acids, including acetic, propionic, butyric, and valeric organic acids, as well as hydrochloric acid. The aim was to determine which acid salts influence the antibacterial and antifungal activity of chitosan-based materials. Two methods were used to modify (formation of ammonium salts) the chitosan nonwovens: First, acid vapors (gassing process) were used to find which salt of chitosan had the best antibacterial properties. Based on the results, the most effective acid was prepared in a solution in ethanol. The influence of the acid concentration in ethanol, the time of treatment of chitosan materials with acid solution, and the rinsing process of modified nonwovens on the antimicrobial activity of the modified materials was investigated. The modified materials were subjected to microbiological tests. Each of the modified materials was placed in bacterial inoculum. The cultures were tested on agar to observe their microbial activity. Toxicity to human red blood cells was also investigated. A reduction in the number of bacterial cells was observed for the *S. aureus* strain with chitosan salt modified with 10% acetic acid in ethanol. The antibacterial activity of the chitosan salts increased with the percentage of acid salts formed on the surface of the solid material (decreasing numbers of bacterial colonies or no growth). No reduction in growth was observed for the *E. coli* strain. The chitosan samples were either inactive or completely eliminated the bacterial cells. Antimicrobial activity was observed for chitosan salts with hydrochloric acid and acetic acid. Finally, ^1^H-NMR spectroscopy and FTIR spectroscopy were used to confirm the incorporation of the acid groups to the amino groups of chitosan.

## 1. Introduction

Chitin is one of the most common polysaccharides in the environment. It can be found in the structures of sponges and corals, as well as in the shells of marine invertebrates and the cell walls of insects and fungi [1,2,3,4,5,6,7]. It was isolated from fungi in 1811 by H. Braconnot, and its structure was described by A. Hofmann [8,9]. Chitosan (CS) is a biopolymer derivative of chitin. It is widely available in various forms (such as films and nonwovens), and is used in numerous industries [10]. It can be obtained by chemical or enzymatic deacetylation. The difference between chitin and CS lies in the degree of deacetylation [11]. The presence of amino and hydroxyl groups in CS makes it a biofunctional polymer that can be selectively modified. Due to its non-toxicity, antibacterial activity, bioadhesive characteristics, biodegradability, and excellent biocompatibility, CS is widely used in biomedical applications, as a drug carrier, antimicrobial, antioxidant, antitumor, and wound-dressing agent [12,13,14,15,16,17,18,19,20,21,22]. 

The mechanisms of action of CS on bacteria and fungi are well known. The main antimicrobial activity of CS is via electrostatic interactions between the cationic groups of CS and negatively charged cell walls [23,24,25,26,27,28,29,30,31,32,33]. Gram-positive and Gram-negative bacteria have differently structured cell walls. Gram-positive bacteria have more peptidoglycans, while Gram-negative bacteria are richer in lipopolysaccharides. These differences can affect the adherence of bacteria to the surface of the materials [34,35]. CS is a polysaccharide composed of glucosamine and acetylglucosamine units with unique physicochemical behavior. The amount of glucosamine residues depends on the degree of chitin deacetylation. CS has the ability to obtain a large number of free NH_3_^+^ ions under acidic conditions, which affects its ability to interact with pathogens. The macromolecules must have a high ionic charge potential to penetrate through the cell membrane. Moreover, the protonation of the amino groups in chitosan influences the possibility of obtaining lipophilic–hydrophilic properties of CS (the polar part consists of NH_3_^+^ and OH groups, as well as the hydrophilic part of the main biopolymer chain). Moreover, the low charge density at neutral and basic pH leads to low solubility, aggregation, and poor stability of chitosan-based formulations [36]. Chitosan itself chelates metal ions—especially those of transition metals—and also finds application as a matrix for the immobilization of enzymes [37].

The antibacterial activity of CS may also be related to the size of the CS molecules. Its potential antimicrobial effects include preventing nutrients from being taken up, and altering cell permeability. High-molecular-weight CS is unable to penetrate the cell wall and cell membrane. Low-molecular-weight CS has both extracellular and intracellular antimicrobial activity [12,38,39,40,41,42]. In addition, molecular weight affects the rate of dissolution of chitosan in acid as the molecular weight increases [43]. The degree of N-acetylation of chitin and CS is an important parameter determining their physicochemical properties. CS modification is related to the degree of acetylation, which is itself related to the number of N-acetylamino groups in the molecules [44]. 

The antimicrobial properties of CS can be improved by chemical modification of the CS structure. The two reactive groups -NH_2_ and -OH offer vast opportunities for chemical modification. These groups allow the formation of several functional derivatives via reactions such as sulfonation, amination, and carboxymethylation [45,46,47]. Hydrophilic, positively charged groups (NH_3_^+^) cause the death of bacterial cells via interaction with and penetration through the cytoplasmic membrane in bacteria [48]. Quaternization improves the positive charge density of CS, and can also improve its antimicrobial efficacy, by enhancing its polycationic nature [32]. Previously, CS has been used in various forms, e.g., as composites, or in various solutions [49,50,51,52].

It is widely believed that chitosan has bacteriostatic properties due to the ammonium salt formation capacity of the biopolymer amino groups. However, much of the literature assumes that chitosan itself inhibits the growth of bacteria.

**Figure 1:**
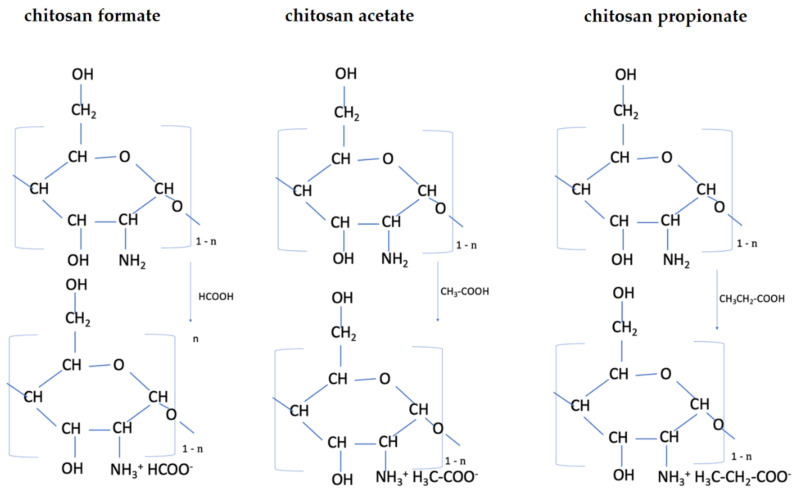
Example of the formation of chitosan salts in the presence of different carboxylic acids, where n is the degree of deacetylation (n = DA).

The aim of this study was to conduct tests on the preparation of chitosan nonwovens modified with various acids—including acetic, propionic, butyric, and valeric organic acids, as well as hydrochloric acid—and to determine which chitosan salts have antibacterial and antifungal activity. Two methods of modifying the chitosan nonwovens were planned: First, acid vapors (gassing process) were used in order to select the salt of chitosan with the best antibacterial properties. Based on the results, the most effective acid was used for research on the protonation of nonwovens reaction examples are shown in Figure 1 (formation of ammonium salts) in solution (wet method). It was assumed that an ethanolic acid solution would be used to eliminate the solubility of the chitosan salt in water. The modified materials were then subjected to microbiological tests (*E. coli* and *S. aureus*). It was also assumed that studies on the influence of modified materials on the process of hemolysis would be performed, which are particularly important from the point of view of antibacterial compounds/materials, as they quite often have adverse hemolytic activity.

## 2. Materials and Methods 

The medical-grade chitosan fibers used in this study were a commercial product of Hismer Biotechnology Co. Ltd., Shandong, China. The deacetylation degree of the used polymer was 91.8. Fibers of 2.02 dtex × 30 mm with a strength of 12cN/tex and relative elongation of 1.5% were used. The nonwoven structure of the biodegradable chitosan fibers was produced using a carder machine and then needled. The final product obtained from the elementary fleeces had a surface weight of 120 g/m^2^ per sheet.

### 2.1. Modification of Chitosan Nonwovens in Different Evaporated Acid Environments 

All acids were sourced from Avantor Performance Materials Poland POOCH Polish Chemicals Reagents, Gliwice, Poland. Hydrochloric (35–38% PURE P.A.), acetic (99.5–99,9% PURE P.A.), formic (98–100% PURE P.A.), propionic (99.5% PURE P.A.), butyric (PURE), and valeric acids (99%) were used to modify the chitosan nonwovens. The procedure for modification was as follows: First, a Petri dish with a diameter of 20 cm containing 20 mL of acid was placed in a desiccator of 3 dm^3^ capacity. This system created an environment saturated with acid vapor after 24 h. The chitosan nonwovens were placed in the acid environments at the same time and removed at 15 min intervals for up to 120 min. The samples after treatment in the acid atmospheres were next placed in a desiccator for 24 h over solid potassium hydroxide granules (PURE P.A. POOCH Polish Chemicals Reagents, Gliwice, Poland) to remove the residual acid condensed on the nonwoven surface. They were then degassed under a vacuum pump. Finally, the nonwoven samples were dried in a normal oven at 40 °C for 2 h. The reaction process in the desiccator was based on the condensation of acid vapors on the surface of the nonwovens, whereupon a chemical reaction occurred between the acid molecules and chitosan.

### 2.2. Modification of Chitosan Nonwovens Using Acetic Acid Solution in Ethanol—Procedure with Rinsing

The following solutions of acetic acid dissolved in ethanol were prepared: 5%, 10%, and 15%. Then, 12 nonwoven samples with dimensions of 5 × 5 cm^2^ and a weight of 1 g were added to a beaker, into which 200 mL of each solution was poured. The samples were submerged for intervals of 10 min for up to 60 min. After removal, some fleece samples were rinsed twice for 5 min with fresh ethanol, then blotted on filter paper and placed on a new filter for 24 h until they were completely dry. This procedure rinsed off any excess solution. Additionally, the nonwoven was dried in an oven at 40 °C for 2 h. The entire procedure was repeated for each of the solutions.

### 2.3. Modification of Chitosan Nonwovens Using Acetic Acid Solution in Ethanol—Procedure without Rinsing

The procedure without rinsing was the same but, after removal, the fleece samples were not rinsed twice for 5 min with fresh ethanol.

### 2.4. Titration Procedure 

A standard solution of hydrochloric acid (0.1 M) and sodium hydroxide (0.1 M) was prepared. Next, 50 mL of sodium hydroxide was poured into a beaker, and 0.1 g of each nonwoven was added. The unreacted sodium hydroxide residue was titrated with an automatic pipette by adding 1 mL of hydrochloric acid every 2 min until it stabilized. The amount of acid introduced was then calculated from the conductometric diagram. The same procedure was performed for all of the samples.

Retention volume was determined using the following formulae:

Number of adsorbed moles of acid (mol) per:

(1) $$n_{x}=n_{NaOH}-n_{HCL}\;(\mathrm{mol})$$

where:

*nNaOH—C x V;*
*nHCL—C x V.*

Number of attached acid groups on nonwovens:

(2) $$AAG=\frac{n_{x}}{m}$$

where:

*n_x_*—number of adsorbed moles of acid (mol/g);
*m*—mass of the nonwoven fabric.

Retention volume:

(3) RV=nxm M chitosan×100% 

where:

*n_x_*—number of adsorbed moles of acid (mol/g);
*m*—mass of nonwoven fabric;
*M*_chitosan_—molar mass of chitosan.

### 2.5. Analysis of Acid Chitosan Salt Forms Based on FTIR Spectra

The molecular structures of the chitosan nonwovens were analyzed using a Nicolet 6700 FTIR spectrometer (Thermo Scientific, Waltham, MA, USA) in attenuated total reflectance (ATR) mode, with a diamond crystal (incidence angle 45°). The measurement conditions were as follows: the resolution was 2 cm^−1^, the range of infrared (IR) radiation was 4000–600 cm^−1^, and 32 scanning procedures of each sample were carried out using IR radiation to obtain the absorption spectra. The absorption spectra of the fiber preparations under investigation were plotted in the system A = f(1/l), as a basis for the interpretation of the molecular nonwoven structure.

The Equations (4) and (5) were used to determine the degree of acetylation (*DA*). Thermo Scientific^TM^ OMNIC^TM^ Specta Software (Thermo Scientific, Waltham, MA, USA) was used for the calculations according to the data [53].

(4) $$DA\left(\%\right)=\frac{A_{1655}}{A_{3450}}\times 100/1.33$$

(5) $$DA\left(\%\right)=\left(\frac{A_{1320}}{A_{1410}}-0.03822\right)/0.03133$$

### 2.6. Analysis of Acid Chitosan Salts Based on NMR Spectroscopy

The chemical structures and DDA (degree of deacetylation) of the initial chitosan, as well as of the chitosan after acid treatment, were determined by ^1^H-NMR spectroscopy analysis using a Bruker AM 400 (Bruker Corporation, Billerica, MA, USA) spectrometer, in a mixed solvent containing DCl (deuterium chloride) and D_2_O (deuterium oxide) (1%, *w*/*w*), with DSS (sodium trimethylsilylpropanesulfonate) as a reference. The samples were prepared as follows: 50 mg of the chitosan salts was dispersed in D_2_O and, after homogenization, was dissolved by adding DCl to finally obtain 1% DCl in H_2_O. When the solution formed, measurements were made at 303 K using 32 scan pulse accumulations. The degree of chitosan deacetylation was calculated using the following formula, where DS is the degree of substitution (6):

(6) $$\mathrm{DS}=\frac{\frac{1}{3}AC\;\underline{H}}{\underline{H}2}$$

where:

**Hypothesis** **1** **(H1):** *Signal integration of proton derived from the acidic residue of the carbon atom in the sugar ring of chitosan*.

**Hypothesis** **2** **(H2):** *Signal integration of proton at the second carbon atom in the sugar ring of chitosan*.

### 2.7. Antimicrobial Activity

Modified chitosan samples were cut into 1 cm^2^ pieces. Two microbiological assays were conducted to evaluate the antimicrobial properties of the materials. The protocol was based on the method described previously, with slight modifications [54]. The toxicity of the chitosan was also assessed. All assays were performed in triplicate.

#### 2.7.1. Susceptibility to Microbial Colonization

Non-sterile samples of the chitosan were placed in 24-well flat-bottomed plates containing Mueller–Hinton II broth (for bacteria) or Sabouraud broth (for fungi) to evaluate the resistance of the materials to microbial colonization. The chitosan fragments were incubated for 24 h at 37 °C (to observe the growth of bacteria) or 25 °C (to observe the growth of fungi). After incubation, 10 µL of medium from each well was seeded on the agar plates. After 24 h of incubation, the appearance of colonies was evaluated.

#### 2.7.2. Bactericidal Activity

The second assay was performed similarly to that described above, with the difference that this time bacterial inocula were used. The aim of this study was to check the ability of the material to kill the microorganisms. Chitosan samples were soaked in 0.5 mL of bacterial suspensions with a density of 0.5 McFarland. Two reference strains were used: *Staphylococcus aureus* ATCC 25923, and *Escherichia coli* ATCC 25922. The samples were incubated for 24 h at 37 °C. After incubation, 10 µL of medium from each well was seeded on the agar plates, and the number of colonies was observed.

### 2.8. Hemolytic Activity of the Chitosan Samples

A hemolysis assay was conducted, using some steps from the protocols described previously [55]. Human red blood cells were obtained from fresh blood collected in test tubes, with EDTA as an anticoagulant. The blood was centrifuged and the plasma removed. After rinsing with PBS, a 4% solution of RBCs was prepared. The chitosan fragments were put into 0.5 mL of RBC solution and incubated for 1 h at 37 °C. Controls with 0% hemolysis (erythrocytes with PBS) and 100% hemolysis (erythrocytes with 1% Triton-X) were prepared. After incubation, 100 µL of the solution from each well was transferred to a 96-well plate and centrifuged (1 h, 4 °C, 7 min, 1000× *g*). The supernatants were resuspended in a new plate, and the release of hemoglobin was measured (540 nm).

## 3. Results

### 3.1. Retention Volume 

To determine the amounts of attached acid groups (corresponding to the amount of ammonium groups formed in the chitosan), the reverse (acid–base) titration method was used. Two standard 0.1 M HCl and 0.1 M NaOH solutions were used as a titrant and as an auxiliary substance, respectively. Additionally, for modified chitosan materials, the retention volume was measured. Table 1, Table 2 and Table 3 show only the results for chitosan derivatives for which colony growth was reduced or absent.

After 15 min in an acidic environment caused by the vaporization of different acids, the retention volume for the chitosan acetate and chitosan hydrochloride was above 90% for all of the tested chitosan nonwovens (Table 1). For chitosan hydrochloride, the retention volume was 93%, while for chitosan acetate it was 92%. After 120 min, the retention volume was lower for HCl modified chitosan (85%) than for chitosan acetate (88%). 

Based on the data in Table 1, it can be concluded that in the initial period of the process the surface sorption process and the associated temporary increase in the amount of acid prevailed. After 60 min, there was an insignificant reduction in the amount of acid, which stabilized. This may be related to the establishment of an equilibrium state between the processes of sorption and desorption.

The data present in Table 2 show that the retention volumes for chitosan acetate were between 80% and 94% for all acid solutions in ethanol and different treatment times, in a procedure without rinsing the samples. The highest rate of retention value was in the case where a 10% solution of acetic acid in ethanol was used and the treatment time was 60 min, while the lowest value was in the case of application of 5% CH_3_COOH solution in ethanol and a treatment time of 20 min. 

Table 3 shows the retention volume of chitosan acetate obtained via the treatment with acetic acid when following the procedure including rising. The values of retention volume for chitosan acetate after rinsing the samples were slightly lower compared to the non-rinsing method. The observed values were above 80%. The highest rates of retention were in the cases of application 10% and 15% solutions of CH_3_COOH for 60 min. 

### 3.2. Microbiology and the Influence of Modified Chitosan Materials on the Hemolysis Process and Toxicity to Human Red Blood Cells 

First, a screening method was used to determine the modified nonwovens with the best antimicrobial properties. The fragments were not sterilized, and were immediately placed in liquid media for fungi or bacteria. This made it possible to identify whether the materials were susceptible to colonization by microorganisms. After 24 h of incubation, the samples were seeded on solid media.

**Figure 2:**
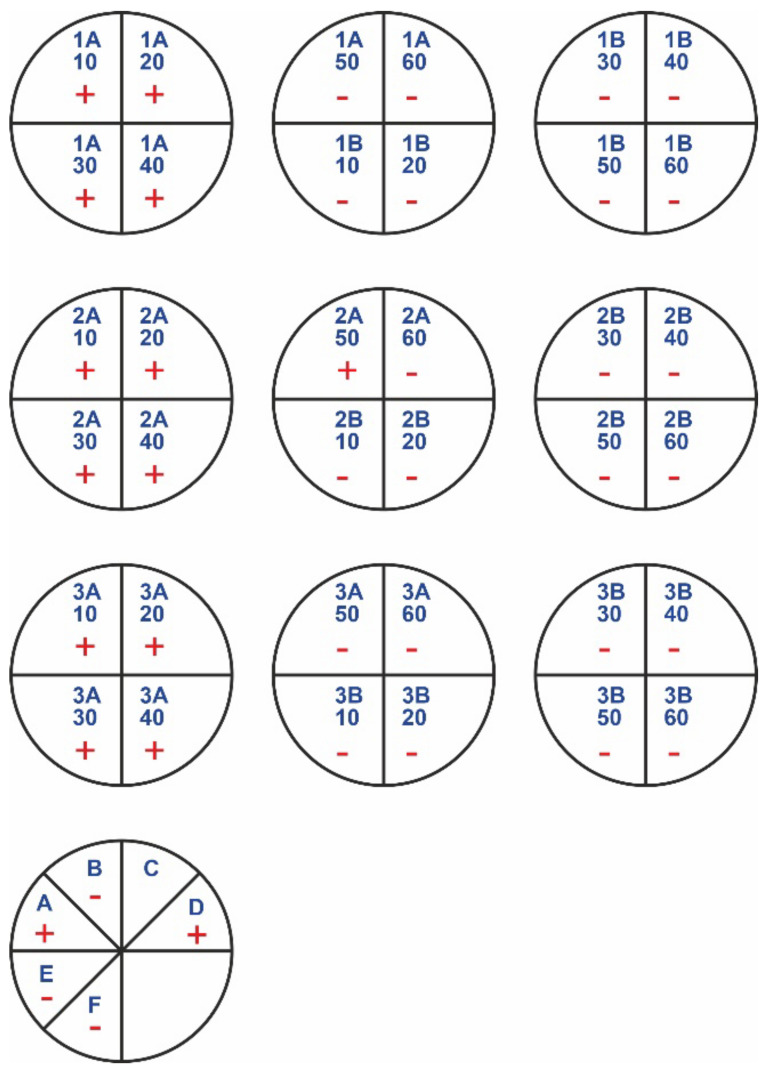
Growth of bacteria in medium (MHA).

**Figure 3:**
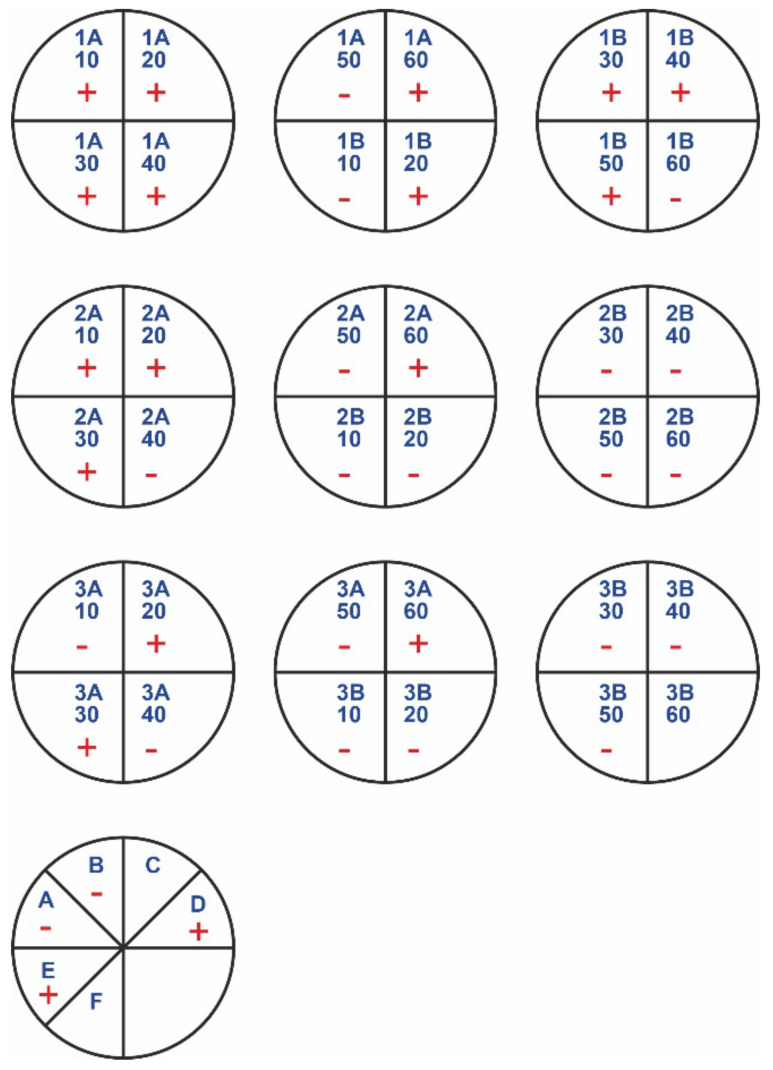
Growth of fungi in medium (SDA).

In Figure 2 and Figure 3, “+” means that bacterial/fungal growth was observed. Mueller–Hinton agar (MHA) was the substrate used for bacteria. Sabouraud agar (SDA) was used as the fungal medium. The letter A indicates the samples with rinsing, while the letter B indicates the samples without rinsing. The samples of nonwovens 1, 2, and 3 correspond to the materials treated with 5%, 10%, and 15% acetic acid in ethanol, respectively. The numbers 10–60 refer to times of treatment, from 10 to 60 min. The most promising results were for the samples with no rinsing, as well as for the samples with rinsing but longer treatment times (50 min or 60 min). In additional observations, a sample of the chitosan hydrochloride was immediately degraded in the liquid medium (it dissolved). 

The last two circles with markings from A to F in Figure 2 and Figure 3 show antimicrobial results of chitosan-based materials treated with vapors of various acids (obtaining modified materials under gassing conditions) (see Table 4).

Activity against bacteria was noted for the samples without rinsing. Therefore, cultures were grown from this group. Growth of organisms was observed in the unmodified blank sample. 

Table 5 shows the effects on microbial growth of modifying the nonwovens using acetic acid solution in ethanol without rinsing. In 10% acetic acid solution in ethanol, the activity of the modified chitosan nonwovens increased against *S. aureus* bacterial cells (decreasing numbers of bacterial colonies or no growth). No reduction in growth was evident for the *E. coli* strain. The chitosan samples were either not active at all, or caused complete elimination of the bacterial cells. 

Antimicrobial activity of the tested materials was observed in acidic atmospheres with hydrochloric acid and acetic acid (Table 6).

Complete absence of hemolysis was noted for materials modified with valeric acid and formic acid. Hemolysis was not observed in the unmodified blank sample. The rinsed nonwovens modified with acetic acid in ethanol solution showed significantly lower impact on hemolysis compared to the non-washed samples (Table 7). Cytotoxicity was noted for samples modified by butyric acid, hydrochloric acid, and acetic acid. The highest toxicity (21.18%) was observed in the case of treatment with acetic acid vapors (Table 8)**.**

### 3.3. Analysis of Obtained Chitosan Salts Based on FTIR–ATR

Changes in the chemical structures of the samples were analyzed using FTIR–ATR spectroscopy. Additionally, the degree of deacetylation was calculated.

Chitosan FTIR spectra showed sharp peaks at 564 cm^−1^ (out-of-plane bending NH, out-of-plane bending C–O), 711 cm^−1^ (out-of-plane bending NH), 1174 cm^−1^ (C–O–C stretching), 2871 cm^−1^ (CH_2_ stretching), and 3430 cm^−1^ (−OH stretching). The vibrational mode of amide C=O stretching was observed at 1650 and 1590 cm^−1^. 

**Figure 4:**
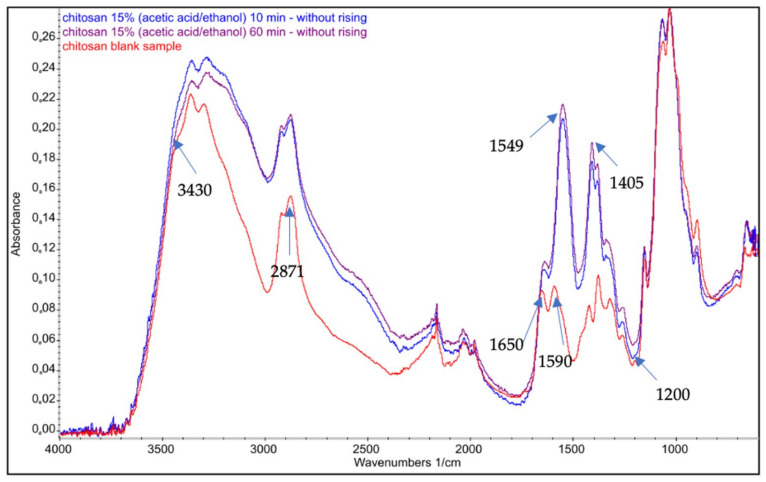
Example FTIR–ATR spectra for **a** nonwoven treated with acetic acid vapor without rinsing.

The spectra for the samples of chitosan nonwovens treated with acetic acid vapor are presented in Figure 4. Based on the spectra, the degree of deacetylation was calculated using Equations (4) and (5) proposed by Czechowska-Biskup et al. [53]. According to the first equation, the degree of deacetylation was 87%, and according to the second it was 89.7%. As can be seen in the spectra, there are differences confirming the formation of chitosan, in the form of a salt derivative of acetic acid, after 10–60 min of exposure to organic acid in the gas phase. There is a strong signal for carboxyl groups, with maxima at 1550 cm^−1^ and 1400 cm^−1^. Adsorption of vaporized acid may occur as result of the interaction of hydrogen bonds between the hydroxyl groups of chitosan and the carboxylic group of the organic acid. After 1 h of exposure to acetic acid vapor, a peak appears with a maximum between 1600 cm^−1^ and 1200 cm^−1^, probably due to the formation of the ammonium salt group of acetic acid and chitosan. There are strong signals for carboxyl groups, with maxima at 1549 and 1405 cm^−1^. We assume the formation of the amine salt of acetic acid at signal 1549 cm^−1^. Signals located in the range of 1410–1340 cm^−1^ are due to the COO^-^ stretching bonds in the acid residue of acetic acid.

### 3.4. Analysis of Obtained Chitosan Salts Based on NMR Spectroscopy

**Figure 5:**
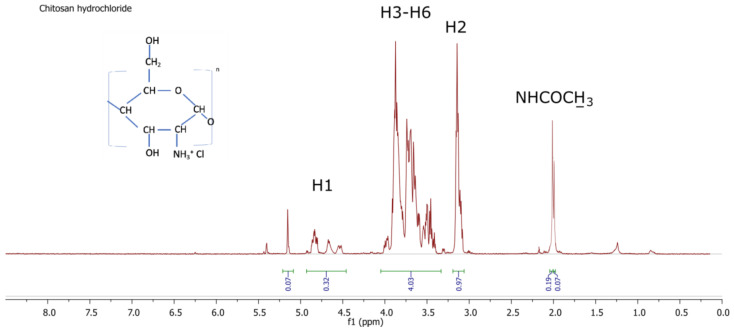
^1^H-NMR spectrum of chitosan hydrochloride.

**Figure 6:**
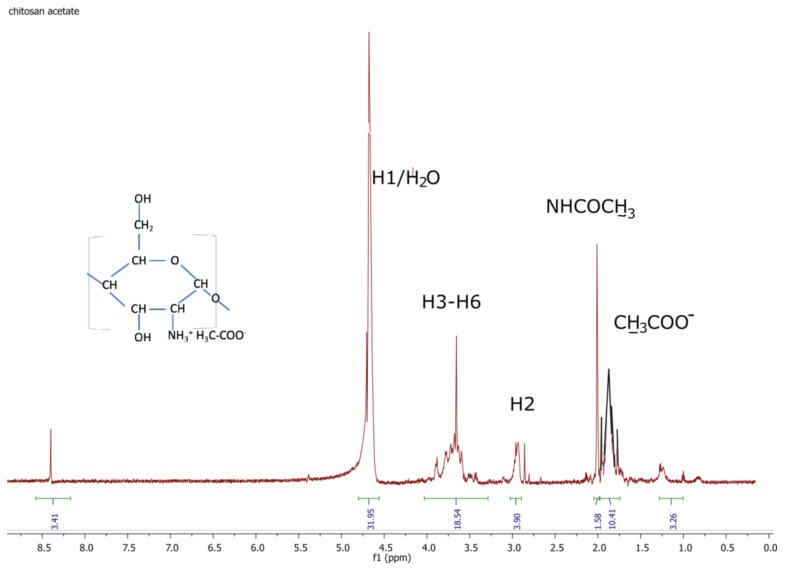
^1^H-NMR spectrum of chitosan acetate.

Figure 5 and Figure 6 show examples of ^1^H-NMR spectra for chitosan-based materials modified with different acids.

On the ^1^H-NMR spectrum of chitosan hydrochloride, the following proton signals can be seen: in the range of 1.8–2.3 ppm protons of the acetamide group NHOCCH_3_; 3.1–3.2 ppm protons bonded to the second carbon of the sugar ring (H2); in the range of 3.3–4.1 ppm protons bonded to the carbons (C3–C6) of the sugar ring (H3–H6); in the range of 4.4–4.9 ppm protons bonded to the first carbon of the sugar ring (H1).

On the ^1^H NMR spectrum of chitosan acetate, the following proton signals can be seen: in the range of 1.6–1.8 ppm for protons of the acetic acid salt -OOCCH_3_; in the range of 1.8–2.1 ppm for protons of the acetamide group NHOCCH_3_; 2.8–3.1 ppm for protons bonded to the second carbon of sugar ring (H2); in the range of 3.3–4.1 for protons bonded to the carbons (C3–C6) of the sugar ring (H3–H6); in the range of 4.6–4.8 for protons bonded to the first carbon of the sugar ring (H1), which is overlapped with the residues of H_2_O.

As can be seen from the NMR spectra for the selected samples with good antibacterial properties (Figure 5), there were no significant changes in the chitosan spectrum, which appears normal. The proton signal from the HCl chitosan salt’s hydrogen overlaps with the DCl protons. Therefore, the existence of hydrogen in the hydrochloric groups cannot be detected. In Figure 6, there is a signal from CH_3_COO^-^, which can be used to calculate and compare the degree of substitution. Table 9. Shows degree of substitution for chitosan salts.

## 4. Discussion

The purpose of this study was to determine the bacteriostatic and bacteriological properties of nonwoven fibrous materials. In a study by Abadehie et al., chitosan/PEO nanofibers mixed with lawsone obtained by electrospinning showed improved wound-healing properties, further demonstrating biocompatibility and antibacterial properties [56]. The retention volumes of the chitosan-based materials modified with various acids show that the acids’ residues may have been incorporated into the structures of the chitosan nonwovens. As can be seen in Table 1, acid vapors were deposited on the surface and within fibers. After some time, degassing occurred, and some of the residues were released from the fibers. This might be related to the uneven structure of the tested materials. At the end of the process, gas equilibrium was obtained. This may be related to the establishment of an equilibrium state between the sorption and desorption processes.

The chitosan nonwovens modified by acetic acid solution in ethanol without rinsing showed a much higher degree of substitution than the nonwovens obtained via the process with a rinsing step. The retention volume of the chitosan acetate was above 80% for both procedures (with and without the rinsing process), but the retention volume was between 10% and 15% higher in the case of the procedure without rinsing. This is probably related to the rinsing process, during which most of the acid was flushed out of the surface. Generally, the higher concentration of acetic acid and extended interaction time led to materials with a higher number of attached acid groups. This was especially noticeable in the case of the rinsed nonwovens modified with 5% and 10% acid solutions in ethanol, for which we observed an increase of 5% in retention volume. In the case of the non-rinsed fabrics, the increase was not so noticeable, because the excess acid residues remained on the surface of the modified material. 

Microbiological tests were used to investigate the activity of the tested materials and the growth of the fungi and bacteria, which were related to the retention volume of the modified nonwovens. Only materials modified with acetic acid and hydrochloric acid were found to have bacteriostatic properties, as can be seen in Table 6. This is probably due to the acid strength and size of the substituents in organic acids adhered to the surface of the nonwoven fabric. Some residues may have been smaller due to the irregular structure of the nonwoven fabric, which has microcapillaries inside it that are not uniformly distributed. 

Activity against bacteria was noted for the samples without rinsing (Table 5). This is because most of the acid residues were on the surface. However, it does not change the fact that only the salts of chitosan with acetic acid and hydrochloric acid have antibacterial properties. In the case of samples with rinsing, the antibacterial effects of the modified chitosan materials were also observed (Figure 2), but only for chitosan nonwovens with longer exposure times to acetic acid and hydrochloric acid.

The results for toxicity to human red blood cells measured as a percentage of hemolysis, presented in Table 7 and Table 8, show much higher toxicity for the samples without rinsing. The highest toxicity was 21.18% for the sample treated with acetic acid. Complete absence of hemolysis was observed for the samples treated with valeric acid and formic acid.

The spectrogram in Figure 4 shows that the modification process did not change the degree of deacetylation in the chitosan nonwovens. FTIR–ATR spectra showed changes in the structure of the material, which confirmed that formation of the salt had taken place. Similar changes in the structure of chitosan can be seen in the ^1^H-NMR spectra shown in Figure 5 and Figure 6. 

A major limitation of the preparation of antimicrobial (antibacterial and antifungal) bacteriostatic chitosan-based materials is obtaining them in the form of suitable acid salts. This limitation is due to the fact that using fibers treated with acids before producing the final form of the material results in complete dissolution of the chitosan salt in the medium in which bacteria are present. Therefore, the use of treatment of chitosan-based materials with acid after preparation of the final material form allows for obtaining materials where only the surface of the chitosan-based matrix is in the form of a biologically active salt. The surface conversion of chitosan amino groups to ammonium salts can be accomplished either through vapors of appropriate acids or the application of alcoholic acid solutions, leaving the core of the material in an insoluble form. The method based on the application of alcoholic acid solutions is safer from the processing point of view. However, when treated with alcoholic solutions, the morphology of the structure can be lost. With wet curing, even with an alcohol solution inert to the fibers, a reduction in fluffiness is observed, which is not observed when treated with acid vapor.

A similar study was conducted by Chung et al. [57], but using aqueous acid solutions. To investigate the antimicrobial activity, the pH was correlated with the properties of the used acids. Acids with higher pKa showed significantly better activity than those with lower pKa in control sets. As found, the application of acids in which the chitosan was dissolved was also an important factor. The best antibacterial activity was found for salts of chitosan and formic acid [57]. This observation indicates that the final antibacterial effect of chitosan derivatives is also influenced by the method of their preparation. In the conditions of chitosan gassing with formic acid vapors, we obtained chitosan formate with moderate antibacterial activity.

Chitosan has a strong affinity toward metal ions because of the presence of numerous amine and hydroxyl groups. Below pH 6.5, chitosan can interact with the bacterial cell wall to destabilize it and alter cell permeability. As was shown by Tripathi et al., chitosan–silver oxide nanocomposite films have good bacteriostatic properties against various kinds of pathogens [58]. Most studies of chitosan in the literature are related to solids and solutions [57,58,59]. 

We used a simple procedure to modify the fibers in the nonwovens, permitting us to obtain new materials with the desired antimicrobial properties and that are soluble in water. We believe that in the case of the obtained materials, polar, positively charged ammonium groups (NH_3_^+^) cause bacterial cell death by interacting with microorganisms and penetrating the bacterial cytoplasmic membrane. Protonation of the amino group with various acids improves the positive charge density of CS, and may also improve its antimicrobial efficacy by enhancing its polycationic nature, which is consistent with the literature data [32,45,46,47,48].

## 5. Conclusions

The research presented in this article enables the effective combination of acids and chitosan nonwovens to achieve antibacterial and antifungal effects, by adjusting the parameters of the solution (concentration of acid and time of treatment). No significant changes were observed in the retention volumes of the samples, showing that all of the acids were well incorporated into the nonwoven structure. Better activity against bacteria was noticed in the case of the samples obtained via procedures using an ethanolic solution of organic acid without rinsing. Therefore, cultures were grown from this group. Growth was also observed in an unmodified blank sample. The best results were obtained for materials treated with vapors of hydrochloric acid and acetic acid, and for the chitosan nonwovens modified using an acetic acid solution in ethanol without rinsing. Only these two chitosan salts showed antimicrobial properties. This was probably due to the acid strength and the size of the adhered acid residues, or the structure of the nonwoven fabric. Some residues may have been smaller due to the irregular structure of the nonwoven fabric, which has channels inside it that are not uniformly distributed. H^1^-NMR and FTIR–ATR spectra confirmed the modification of the chitosan.

We intend to perform this study for other forms of chitosan, such as scaffolds, foils, and chitosan microspheres, because we expect that the method of formation of ammonium salts of chitosan in its final forms developed by us may be of a general nature, thanks to which it will be possible to obtain various chitosan materials with antibacterial properties. 

## Figures and Tables

**Table 1:** The retention volume of modified chitosan nonwovens modified by acid fumes (gassing process).

Time of Incubation of Chitosan Material in Acid Vapor (min)	Retention Volume (%) after Treatment with Gaseous HCl	Retention Volume (%) after Treatment with CH_3_COOH Vapor
15	93	92
60	86	87
120	85	88

**Table 2:** The retention volume of modified chitosan nonwovens after treatment in solutions of acetic acid in ethanol—procedure without rinsing.

Sample	Retention Volume (%)	Sample	Retention Volume (%)	Sample	Retention Volume (%)
5/10	88	10/10	95	15/10	92
5/20	81	10/20	92	15/20	93
5/30	82	10/30	90	15/30	94
5/40	80	10/40	93	15/40	93
5/50	87	10/50	93	15/50	94
5/60	89	10/60	95	15/60	94

5: acetic acid concentration in ethanol; 10–60: treatment time (min) of nonwovens in a bath of acetic acid in ethanol.

**Table 3:** The retention volume of modified chitosan nonwovens after treatment in solutions of acetic acid and ethanol—procedure with rinsing.

Sample	Retention Volume (%)	Sample	Retention Volume (%)	Sample	Retention Volume (%)
5/10	85	10/10	86	15/10	90
5/20	86	10/20	85	15/20	86
5/30	88	10/30	84	15/30	88
5/40	90	10/40	89	15/40	89
5/50	92	10/50	91	15/50	91
5/60	91	10/60	92	15/60	92

5: acetic acid concentration in ethanol; 10–60: time (min) of nonwovens in a bath of acetic acid and ethanol.

**Table 4:** Descriptions of the letters on the tested Petri dishes.

Marks on Circles	Acid Atmosphere
A	Valeric acid
B	Propionic acid
C	Formic acid
D	Butyric acid
E	Hydrochloric acid
F	Acetic acid

**Table 5:** Effects of modification of chitosan-based materials on microbial growth for nonwovens modified using acetic acid solution in ethanol—procedure without rinsing.

Sample	*S. aureus* ATCC 25923	*E. coli* ATCC 25922
5/10	Growth	Growth
5/20	Growth	Growth
5/30	Growth	Growth
5/40	Growth	Growth
5/50	Growth	Growth
5/60	Growth	Growth
10/10	Growth: visible growth reduction	Growth
10/20	Growth: 170 * colonies	Growth
10/30	Growth	No growth
10/40	Growth: 70 * colonies	Growth
10/50	Growth: reduction	Growth
10/60	No growth	Growth
15/10	Growth: 14 * colonies	Growth
15/20	Growth: 12 * colonies	Growth
15/30	Growth: 30 * colonies	No growth
15/40	No growth	No growth
15/50	Growth: 9 * colonies	No growth
15/60	No growth	No growth

*—The mean values of 3 repetitions.

**Table 6:** Effects of modification of chitosan-based materials on microbial growth for nonwovens modified by vapors of various acids (obtaining modified materials under gassing conditions).

Sample	*S. aureus* ATCC 25923	*E. coli* ATCC 25922
Valeric acid	Growth	Growth
Propionic acid	Growth	Growth
Formic acid	Growth: 5 * colonies	Growth
Butyric acid	Growth	Growth
Hydrochloric acid	No growth	No growth
Acetic acid	No growth	No growth

*—The mean value of 3 repetitions.

**Table 7:** Toxicity to human red blood cells measured as % of hemolysis in nonwovens modified using acetic acid solution in ethanol—procedures with and without rinsing.

Samples	% of Hemolysis in Rinsed Samples	% of Hemolysis in Non-Rinsed Samples
5/10	0.24	0.09
5/20	0.45	1.17
5/30	0.80	1.52
5/40	0.36	2.70
5/50	0.13	1.28
5/60	0.54	0.46
10/10	0.00	14.34
10/20	0.26	3.59
10/30	0.16	14.24
10/40	0.00	17.51
10/50	0.65	15.36
10/60	0.82	14.88
15/10	0.47	10.09
15/20	0.26	1.12
15/30	0.55	13.21
15/40	0.16	16.99
15/50	2.01	14.78
15/60	3.21	20.87

**Table 8:** Toxicity to human red blood cells measured as % of hemolysis in nonwovens modified by vapors of various acids (obtaining modified materials under gassing conditions).

Acids	Valeric Acid	Propionic Acid	Formic Acid	Butyric Acid	Hydrochloric Acid	Acetic Acid
% Hemolysis	0.00	0.26	0.00	1.04	0.88	21.18

**Table 9:** Degree of substitution for chitosan salts, calculated based on ^1^H-NMR spectra.

Sample	Degree of Substitution (%)	Degree of Substitution (%) Based on Titration
Chitosan hydrochloride	-	85
Chitosan acetate	88.0	89

## Data Availability

Data are stored at the Institute of Textile Materials and Polymer Composites.

## References

[1] Nature El-Sherbiny I.M., El-Baz N.M., Thakur V.K., Thakur M.K. (2015). A Review on Bionanocomposites Based on Chitosan and Its Derivatives for Biomedical Applications. Eco-Friendly Polymer Nanocomposites.

[2] Ehrlich H. (2010). Chitin and collagen as universal and alternative templates in biomineralization. Int. Geol. Rev..

[3] Juárez-de La Rosa B.A., Quintana P., Ardisson P.L., Yáñez-Limón J.M., Alvarado-Gil J.J. (2012). Effects of thermal treatments on the structure of two black coral species chitinous exoskeleton. J. Mater. Sci..

[4] Sikorski D., Gzyra-Jagieła K., Draczyński Z. (2021). The kinetics of chitosan degradation in organic acid solutions. Mar. Drugs.

[5] Grgac S.F., Tarbuk A., Dekanic T., Sujka W., Draczynski Z. (2020). The chitosan implementation into cotton and polyester/cotton blend fabrics. Materials.

[6] João C.F.C., Silva J., Borges J.P., Thakur V.K., Thakur M.K. (2015). Chitin-based nanocomposites: Biomedical applications. Eco-friendly Polymer Nanocomposites: Chemistry and Applications.

[7] Tyliszczak B. (2016). Animal-derived chitosans. Characteristics, comparison, application Chitozany zwierzęce. Charakterystyka, porównanie, wykorzystanie. Przemysł Chem..

[8] Wysokowski M., Petrenko I., Stelling A.L., Stawski D., Jesionowski T., Ehrlich H. (2015). Poriferan chitin as a versatile template for extreme biomimetics. Polymers.

[9] Khoushab F., Yamabhai M. (2010). Chitin research revisited. Mar. Drugs.

[10] Pokhrel S., Yadav P.N., Adhikari R. (2016). Applications of Chitin and Chitosan in Industry and Medical Science: A Review. Nepal J. Sci. Technol..

[11] Venkatesan J., Kim S.K. (2010). Chitosan composites for bone tissue engineering—An overview. Mar. Drugs.

[12] Goy R.C., De Britto D., Assis O.B.G. (2009). A review of the antimicrobial activity of chitosan. Polimeros.

[13] Young D.H., Köhle H., Kauss H. (1982). Effect of Chitosan on Membrane Permeability of Suspension-Cultured Glycine max and Phaseolus vulgaris Cells. Plant Physiol..

[14] Felt O., Buri P., Gurny R. (1998). Chitosan: A unique polysaccharide for drug delivery. Drug Dev. Ind. Pharm..

[15] Mi F.L., Tan Y.C., Liang H.F., Sung H.W. (2002). In vivo biocompatibility and degradability of a novel injectable-chitosan-based implant. Biomaterials.

[16] Tamer T.M., Valachová K., Hassan M.A., Omer A.M., El-Shafeey M., Eldin M.S.M., Šoltés L. (2018). Chitosan/hyaluronan/edaravone membranes for anti-inflammatory wound dressing: In vitro and in vivo evaluation studies. Mater. Sci Eng. C.

[17] Omer A.M., Tamer T.M., Hassan M.A., Rychter P., Mohy Eldin M.S., Koseva N. (2016). Development of amphoteric alginate/aminated chitosan coated microbeads for oral protein delivery. Int. J. Biol. Macromol..

[18] Yildirim-Aksoy M., Beck B.H. (2017). Antimicrobial activity of chitosan and a chitosan oligomer against bacterial pathogens of warmwater fish. J. Appl. Microbiol..

[19] Valachová K., Tamer T.M., Eldin M.M., Šoltés L. (2016). Radical-scavenging activity of glutathione, chitin derivatives and their combination. Chem. Pap..

[20] Xie F., Ding R.L., He W.F., Liu Z.J.L., Fu S.Z., Wu J.B., Yang L.L., Lin S., Wen Q.L. (2017). In vivo antitumor effect of endostatin-loaded chitosan nanoparticles combined with paclitaxel on lewis lung carcinoma. Drug Deliv..

[21] Archana D., Dutta J., Dutta P.K. (2013). Evaluation of chitosan nano dressing for wound healing: Characterization, in vitro and in vivo studies. Int. J. Biol. Macromol..

[22] Tamer T.M., Collins M.N., Valachová K., Hassan M.A., Omer A.M., Mohy-Eldin M.S., Švík K., Jurčík R., Ondruška Ľ., Biró C. (2018). MitoQ loaded chitosan-hyaluronan composite membranes for wound healing. Materials.

[23] Felt O., Carrel A., Baehni P., Buri P., Gurny R. (2000). Chitosan as tear substitute: A wetting agent endowed with antimicrobial efficacy. J. Ocul. Pharmacol. Ther..

[24] Rabea E.I., Badawy M.E.T., Stevens C.V., Smagghe G., Steurbaut W. (2003). Chitosan as antimicrobial agent: Applications and mode of action. Biomacromolecules.

[25] Ke C.L., Deng F.S., Chuang C.Y., Lin C.H. (2021). Antimicrobial actions and applications of Chitosan. Polymers.

[26] Hu Y., Du Y., Wang X., Feng T. (2009). Self-aggregation of water-soluble chitosan and solubilization of thymol as an antimicrobial agent. J. Biomed. Mater. Res. Part A.

[27] Gómez-Estaca J., López de Lacey A., López-Caballero M.E., Gómez-Guillén M.C., Montero P. (2010). Biodegradable gelatin-chitosan films incorporated with essential oils as antimicrobial agents for fish preservation. Food Microbiol..

[28] Kong M., Chen X.G., Xing K., Park H.J. (2010). Antimicrobial properties of chitosan and mode of action: A state of the art review. Int. J. Food Microbiol..

[29] Hernández-Ochoa L., Gonzales-Gonzales A., Gutiérrez-Mendez N., Muñoz-Castellanos L.N., Quintero-Ramos A. (2011). Study of the antibacterial activity of chitosan-based films prepared with different molecular weights including spices essential oils and functional extracts as antimicrobial agents. Rev. Mex. Ing. Química.

[30] Huang L., Dai T., Xuan Y., Tegos G.P., Hamblin M.R. (2011). Synergistic combination of chitosan acetate with nanoparticle silver as a topical antimicrobial: Efficacy against bacterial burn infections. Antimicrob. Agents Chemother..

[31] Mohamed N.A., Sabaa M.W., El-Ghandour A.H., Abdel-Aziz M.M., Abdel-Gawad O.F. (2013). Quaternized N-substituted carboxymethyl chitosan derivatives as antimicrobial agents. Int. J. Biol. Macromol..

[32] Tan H., Ma R., Lin C., Liu Z., Tang T. (2013). Quaternized chitosan as an antimicrobial agent: Antimicrobial activity, mechanism of action and biomedical applications in orthopedics. Int. J. Mol. Sci..

[33] Avadi M.R., Sadeghi A.M.M., Tahzibi A., Bayati K.H., Pouladzadeh M., Zohuriaan-Mehr M.J., Rafiee-Tehrani M. (2004). Diethylmethyl chitosan as an antimicrobial agent: Synthesis, characterization and antibacterial effects. Eur. Polym. J..

[34] Gan L., Chen S., Jensen G.J. (2008). Molecular organization of Gram-negative peptidoglycan. Proc. Natl. Acad. Sci. USA.

[35] Rohde M. (2019). The Gram-Positive Bacterial Cell Wall. Microbiol. Spectr..

[36] García O.G.Z., Oropeza-Guzmán M.T., Argüelles Monal W.M., López-Maldonado E.A. (2019). Design and mechanism of action of multifunctional BPE’s with high performance in the separation of hazardous metal ions from polluted water Part I: Chitosan-poly(N-vinylcaprolactam) and its derivatives. Chem. Eng. J..

[37] Dutta P.K., Duta J., Tripathi V.S. (2004). Chitin and Chitosan: Chemistry, properties and applications. J. Sci. Ind. Res..

[38] Zubareva A., Shagdarova B., Varlamov V., Kashirina E., Svirshchevskaya E. (2017). Penetration and toxicity of chitosan and its derivatives. Eur. Polym. J..

[39] Kravanja G., Primožič M., Knez Ž., Leitgeb M. (2019). Chitosan-based (Nano)materials for Novel Biomedical Applications. Molecules.

[40] Sudarshan N.R., Hoover D.G., Knorr D. (1992). Antibacterial Action of Chitosan. Food Biotechnol..

[41] Devlieghere F., Vermeulen A., Debevere J. (2004). Chitosan: Antimicrobial activity, interactions with food components and applicability as a coating on fruit and vegetables. Food Microbiol..

[42] Shagdarova B.T., Il’ina A.V., Varlamov V.P. (2016). Antibacterial Activity of Alkylated and Acylated Derivatives of Low–Molecular Weight Chitosan. Appl. Biochem. Microbiol..

[43] Panda P.K., Yang J.M., Chang Y.H., Su W.W. (2019). Modification of different molecular weights of chitosan by p-Coumaric acid: Preparation, characterization and effect of molecular weight on its water solubility and antioxidant property. Int. J. Biol. Macromol..

[44] Aranaz I., Mengíbar M., Harris R., Paños I., Miralles B., Acosta N., Galed G., Heras Á. (2012). Functional Characterization of Chitin and Chitosan. Curr. Chem. Biol..

[45] Rwei S.P., Lien C.C. (2014). Synthesis and viscoelastic characterization of sulfonated chitosan solutions. Colloid Polym. Sci..

[46] Amir Afshar H., Ghaee A. (2016). Preparation of aminated chitosan/alginate scaffold containing halloysite nanotubes with improved cell attachment. Carbohydr. Polym..

[47] Bukzem A.L., Signini R., dos Santos D.M., Lião L.M., Ascheri D.P.R. (2016). Optimization of carboxymethyl chitosan synthesis using response surface methodology and desirability function. Int. J. Biol. Macromol..

[48] Jiang S., Wang L., Yu H., Chen Y. (2005). Preparation of crosslinked polystyrenes with quaternary ammonium and their antibacterial behavior. React. Funct. Polym..

[49] Xu J., Manepalli P.H., Zhu L., Narayan-Sarathy S., Alavi S. (2019). Morphological, barrier and mechanical properties of films from poly (butylene succinate) reinforced with nanocrystalline cellulose and chitin whiskers using melt extrusion. J. Polym. Res..

[50] Singh A., Dutta P.K., Kumar H., Kureel A.K., Rai A.K. (2019). Improved antibacterial and antioxidant activities of gallic acid grafted chitin-glucan complex. J. Polym. Res..

[51] Panda P.K., Yang J.M., Chang Y.H. (2021). Preparation and characterization of ferulic acid-modified water soluble chitosan and poly (γ-glutamic acid) polyelectrolyte films through layer-by-layer assembly towards protein adsorption. Int. J. Biol. Macromol..

[52] Panda P.K., Dash P., Yang J.M., Chang Y.H. (2022). Development of chitosan, graphene oxide, and cerium oxide composite blended films: Structural, physical, and functional properties. Cellulose.

[53] Czechowska-Biskup R., Jarosińska D., Rokita B., Ulański P., Rosiak J.M. (2012). Determination of degree of deacetylation of chitosan-Comparision of methods. Prog. Chem. Appl. Chitin Its Deriv..

[54] Maciejewska M., Bauer M., Neubauer D., Kamysz W., Dawgul M. (2016). Influence of amphibian antimicrobial peptides and short lipopeptides on bacterial biofilms formed on contact lenses. Materials.

[55] Avrahami D., Shai Y. (2004). A new group of antifungal and antibacterial lipopeptides derived from non-membrane active peptides conjugated to palmitic acid. J. Biol. Chem..

[56] Abadehie F.S., Dehkordi A.H., Zafari M., Bagheri M., Yousefiasl S., Pourmotabed S., Mahmoodnia L., Validi M., Ashrafizadeh M., Zare E.N. (2021). Lawsone-encapsulated chitosan/polyethylene oxide nanofibrous mat as a potential antibacterial biobased wound dressing. Eng Regen..

[57] Chung Y.C., Wang H.L., Chen Y.M., Li S.L. (2003). Effect of abiotic factors on the antibacterial activity of chitosan against waterborne pathogens. Bioresour. Technol..

[58] Tripathi S., Mehrotra G.K., Dutta P.K. (2011). Chitosan-silver oxide nanocomposite film: Preparation and antimicrobial activity. Bull Mater. Sci..

[59] Yilmaz Atay H., Jana S., Jana S. (2020). Antibacterial Activity of Chitosan-Based Systems. Functional Chitosan: Drug Delivery and Biomedical Applications.

